# Social value framing of physical activity in European Member State policies: a content analysis

**DOI:** 10.3389/fspor.2024.1415007

**Published:** 2024-06-05

**Authors:** I. Ritchie, I. Nieto, M. Brunn, X. Mayo, A. Jimenez

**Affiliations:** ^1^ Master of Public Health Program, École des Hautes Études en Santé Publique (EHESP), Paris, France; ^2^ THiNKactive Research Centre, EuropeActive, Brussels, Belgium; ^3^ Sports Science Research Centre, King Juan Carlos University, Madrid, Spain

**Keywords:** physical activity, social value, policy, multisectoral action, issue framing

## Abstract

**Background:**

Engagement in physical activity (PA) benefits physical and mental health as well as many other areas of society. In Europe however, 1/3 adults do not meet minimum PA recommendations. Social value, and its quantification through social return on investment (SROI) evidence, may be a useful framing to enhance PA promotion. This study aimed to assess the current use of social value framing of PA in European Union (EU) policies.

**Methods:**

Content analysis of 45 EU member state policies which contain reference to PA was conducted to evaluate the presence of five social value domains and SROI evidence. Data was analysed using manual inductive coding, supported by DeepL translation and NVivo tools.

**Results:**

Social value framing was present to a certain extent in existing policies, with improved health being the most commonly referenced benefit of PA, followed by reference to social and community and then environmental benefits. Acknowledgement of the positive impacts of PA on wellbeing and education was the least present. Reference to SROI evidence was also limited. Generally, policies lacked holistic recognition of the social value of PA. Policies from the health sector were particularly limited in recognising the wider benefits of PA, whilst those from the environmental sector acknowledged the widest range of co-benefits.

**Conclusion:**

Adopting social value framing could be a useful approach for enhancing PA promotion. Whilst it is present to a certain extent in existing policy, this could be increased in terms of comprehensiveness to increase issue salience and multisectoral policy action.

## Introduction

The World Health Organization (WHO) recommends physical activity (PA) as an essential part of a healthy lifestyle, specifying at least 150–300 min/week of moderate-intensity aerobic PA for adults and at least 60 min/day of moderate-to-vigorous intensity PA for children (1). In reality however, the world is experiencing a pandemic of physical inactivity (2, 3). In Europe, over a third of adults do not meet these minimum guidelines, with recent data revealing that only 14% of the population performs PA “regularly”, meaning at least five days per week (4, 5). This situation is highly concerning given the strong negative impact of inactivity on health (6). In the European Union (EU), inactivity is responsible for 1 million deaths per year and is a strong risk factor for non-communicable diseases, such as obesity, type 2 diabetes, and cancer, which cost the EU €115bn annually (5, 7). Crucially, despite inactivity being a *modifiable* risk factor, only 2.8% of total health expenditure across Europe goes to prevention, including PA promotion (5, 8, 9).

PA is a complex behaviour, with engagement influenced by a diverse range of factors. At the individual level, psychological factors such as motivation and both actual and perceived ability to participate are key to understanding behavioural change [see self-efficacy theory (10, 11) and self-determination theory (12)]. At the same time, PA is strongly influenced by interactions between individuals and their socio-environmental context including urban design, active transport infrastructures, perceived safety and social norms (13, 14). Creating conducive physical and social environments which increase the feasibility of engaging in PA and support both intrinsic and extrinsic motivation is essential (15). Multisectoral collaboration for PA promotion holds significant potential for this purpose, defined as collaboration between stakeholders across sectors towards a common aim, at local, regional and national scales (16, 17).

The WHO Global Action Plan on Physical Activity (9) calls for the evaluation and development of different messaging around PA to strengthen policy frameworks, enhance stakeholder engagement and increase its position in political agendas to support multisectoral action. All EU countries have at least one national policy or action plan on PA promotion, with it also present in EU-level policies such as the Tartu Call for a Healthy Lifestyle and the EU Work Plan for Sport 2021–2024 (18–20). Furthermore, as part of the EU Commission's Healthier Together initiative (21), sixteen EU countries endorsed promoting PA as a priority area for non-communicable disease prevention. Despite these efforts, inactivity levels “remain unacceptably high” (7).

Framing analysis helps to identify how an issue is represented in political arenas (22). It can be particularly useful regarding Kingdon's (23) multiple streams framework, which claims that in order for policy action to occur, a policy problem must be specifically defined and matched with a suitable policy response, in a favourable political environment (24, 25). To support this, an issue can be tied in with political priorities and framed to appeal to broader interest groups (26, 27). Recently, the framing of public health issues has started to shift from a focus on individual behaviour to the relevance of the environment surrounding that behaviour (e.g., broader public health interventions promoting exercise vs. personal choice to exercise) (28). The recent WHO Europe (2023) publication “*Making health for all policies: Harnessing the co-benefits of health*” promotes that the wider social, environmental, and economic benefits of interventions should be more explicit in health policies to counter the tendency of sectoral differentiation (29). Importantly, limited studies have been conducted on strategic issue framing in public health hence this is an area where further research is needed (26).

Social value research began in the 1960s due to increasing interest in the impacts of PA beyond health, including both direct impacts on individuals and positive externalities for wider society (30, 31). A contemporary review by Taylor et al. (32) identified five domains of impact of PA: health, crime, education, social capital, and subjective wellbeing. Using this concept for framing PA in policy could be useful for two reasons. Firstly, social value framing highlights the benefits of PA across a wide range of domains (33). This can appeal to diverse stakeholders which supports multisectoral action (17). Secondly, the Social Return on Investment (SROI) model provides a methodology to transform such social value benefits into a monetary ratio (34). For example, Sport England (35) estimated that for every £1 invested in community sport and PA in England in 2017/18, a social return of £3.91 was created. Given that economic quantification can be a highly impactful form of evidence for policymakers (36), the use of SROI may help to enhance the political prioritisation of PA promotion, shifting the narrative from one of cost to one of investment (33).

Considering prevailing physical inactivity in Europe, it is evident that existing PA promotion efforts are limited in their efficacy. It is therefore important to establish robust knowledge of the content of existing policy, notably the current frames being used in relation to PA, and assess whether strategic reframing could maximise progress. To the best of the researchers' knowledge, this is the first study on the use of social value framing in European Member State policies which contain reference to PA.

## Method

Policy content analysis was used as a systematic way to explore framings present in existing policies (37). An audit approach was adopted to provide an overview of the policy environment without judging or grading the evidence (38).

### Sampling

EU member state (*n* = 27) policies with content related to PA were the focus of this analysis. To locate the documents, WHO Europe 2021 Physical Activity Factsheets were searched to identify the titles of relevant policies per country, defined as “written documentation of strategies and priorities with defined goals” (39). Results were then cross-checked with the European Education and Culture Executive Agency's (EACEA) National Policies Platform Section 7.3 “Sport, youth fitness and physical activity” per country (40). New resources identified through the EACEA were added to gain a more comprehensive sample. Inclusion criteria were pre-defined, with documents included if they were; (1) a policy, programme, or action plan, (2) full-text publicly available online in PDF or Word formats, (3) written by a national or regional government or a national association, and (4) published in English or the EU member state's official language. Policies originally published in a language other than English were translated into British English using the computer-assisted translation tool DeepL. One policy included was published in Croatian, a language not currently supported by DeepL. Google Translate was used in this case. There were no constraints regarding the publication date. Educational curriculums and documents published in an informal format (e.g., only available as a webpage) were excluded.

**Figure 1:**
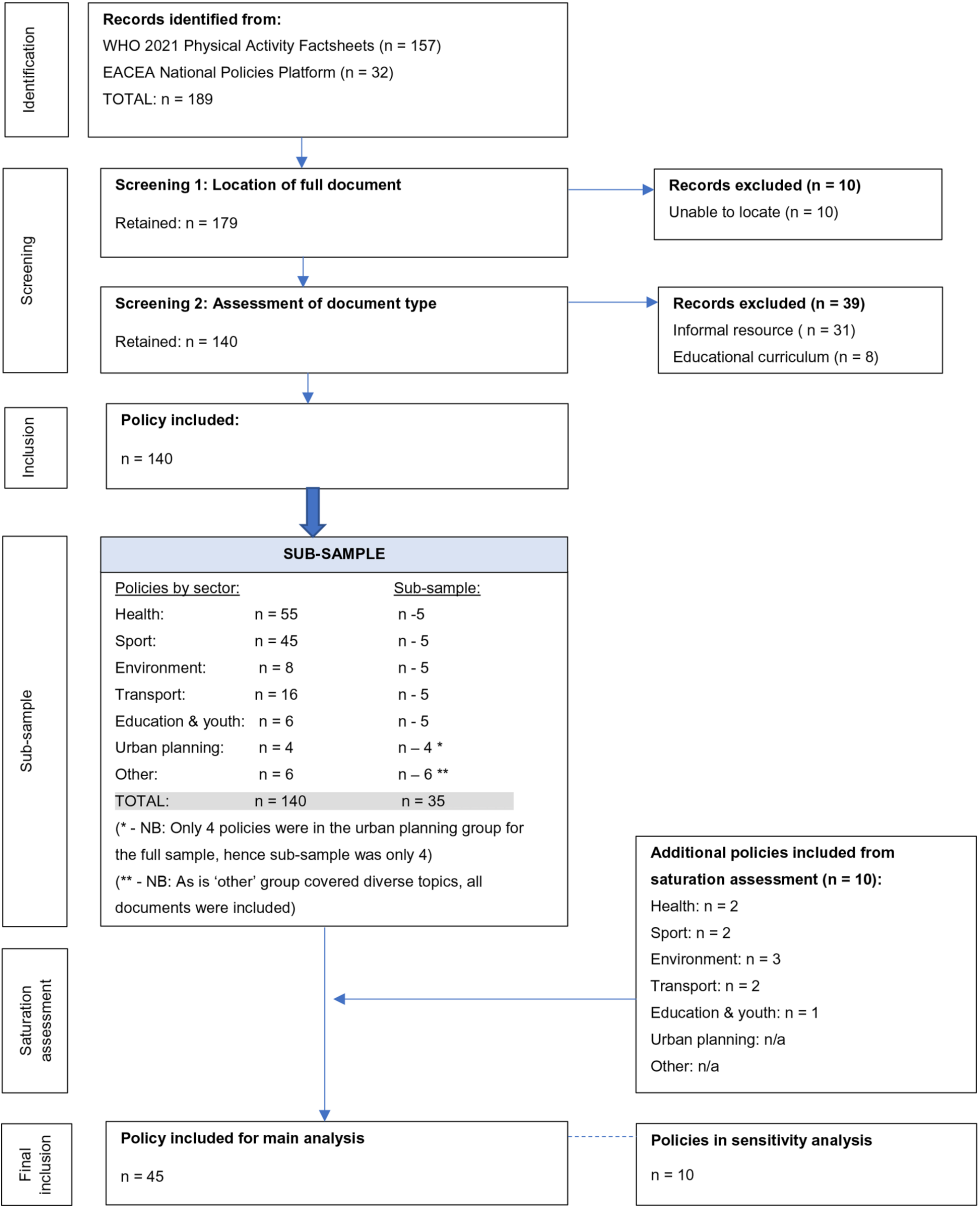
Policy document inclusion flowchart.

A subsample of policies was selected to facilitate analysis given the likelihood of recurring themes in similar policies and the asymptotic curve in qualitative data collection (most new information is identified at the start of the analysis process, with less new information as more analysis events occur) (41). To facilitate this, the included documents were separated into sector groups based on those from the WHO 2021 Physical Activity Factsheets: health, sport, environment, transport, education, and urban planning (39). The education category was expanded to be “education and youth” to facilitate policy grouping. An “other” category was also created for documents which did not naturally fit into any category. The categorisation was firstly based on the author (e.g., Ministry of Health, Ministry of Education). For policies with no specific ministry stated, allocation was based on the title of the document. This was also used where the stated ministry was multisectoral between the sector groups (e.g., Ministry of Health and Sport). Such allocations were guided by previous research on PA policy analysis (38). Finally, the policies were ranked by publication date in each sector group and the five most recent policies selected for the analysis. If the same date was shared by multiple policies, selection was based on alphabetisation by country name (A-Z). Figure 1 shows the flowchart for inclusion of the policies.

### Analysis

The initial coding framework for this study contained six main codes (health, wellbeing, social and community, education, environment, and other), adapted from Taylor et al.'s (32) social domain framework. These modifications were based on literature published since 2015 and wider literature on the social value of PA, rather than only sport. Following this procedure, Taylor et al.'s crime domain was integrated with social capital to form a more comprehensive social and community domain, and a category for the environmental benefits of PA was created.

NVivo version 1.7.1 was used to manually code all policies. An inductive approach was taken to help identify and describe patterns in the data (42). First, content analysis organised the information into categories based on the initial coding framework. Thematic analysis was then used, involving a repeated, iterative process of sub-code creation to identify emergent themes (37).

Saturation assessment was used to assess the point at which analysis of further policies produced limited new information. Guest et al.'s (41) method for saturation testing was used for each subsample sector group. The method involved comparing the themes identified from a base sample and those from additional documents. For this study, a base size of five documents was used for each subsample sector group. Next, a run length of 2 was defined, which is the number of policies within which it is aimed to identify new themes. For each run, the next two most recently published policies in that sector group were analysed, and the identified themes were compared to the base sample. A saturation ratio was calculated, with new items documented in the numerator and the base themes in the denominator. Finally, a new information threshold of 0% was used to assess whether saturation had been reached. For each sector group, if this threshold was not reached, another run was completed until 0% was obtained. Once saturation assessment was complete, the thematic coding of the full subsample was re-assessed to ensure comprehensiveness.

Given that 10 EU countries (Belgium, Cyprus, Czechia, Italy, Latvia, Luxembourg, Malta, Romania, Slovakia, and Slovenia) were not represented in the final subsample of documents, a sensitivity analysis was conducted. The most recent identified policy from each of these countries was selected, with alphabetisation used where dates were shared, to ensure objectivity. An identical analysis process was followed, and the themes identified were compared to those from the included subsample. No new themes emerged, which supports the comprehensiveness of the original analysis.

## Results

### Overview of policy subsample

In total, 45 policy documents were analysed, equating to 23.8% of those initially identified. Thirty-five of these documents were from the original subsample, and a further 10 were added from saturation assessment. Overall, 34 documents were originally identified from the WHO 2021 Physical Activity Factsheets and 11 added from the EACEA National Policies Platform. The full policy list can be found in Supplementary Table S2 in Supplementary Materials, which shows included policies labelled by sector group (e.g., Policy_H for health policies, Policy_E for education policies). Publication dates ranged from 2005 to 2022, with policies coming from Sweden (*n* = 9), Bulgaria (*n* = 4), Poland (*n* = 4), Finland (*n* = 3), Hungary (*n* = 3), Ireland (*n* = 3), Portugal (*n* = 3), Austria (*n* = 2), Croatia (*n* = 2), Denmark (*n* = 2), France (*n* = 2), Greece (*n* = 2), Lithuania (*n* = 2), Estonia (*n* = 1), Germany (*n* = 1), Netherlands (*n* = 1) and Spain (*n* = 1).

Health sector documents included general national health programmes (*n* = 5), one PA-specific plan, and one non-communicable disease plan. The sport sector included general national sports programmes (*n* = 5) and those targeting specific sub-populations (*n* = 2). Environment sector documents included general national plans for environmental protection (*n* = 4), plans for the recreational use of nature (*n* = 3), and one plan on energy usage in the sport sector. For transport, documents included transport infrastructure plans (*n* = 3), cycling plans (*n* = 2), and general mobility plans (*n* = 2). In the education and youth group, documents included general national youth strategies (*n* = 3) and plans focused specifically on youth recreation and school sports (*n* = 3). All four documents from the urban planning sector were related to general urban design, as opposed to those previously mentioned as part of the transport group which focused specifically on transport infrastructures. Finally, the “other” group contained national development strategies (*n* = 3), plans focused on the inclusion of marginalised communities (*n* = 2) and an intersectoral commission policy article (*n* = 1).

### Presence of social value framing

All five social value domains were identified in the sample of documents. Table 1 presents the frequency of domain and sub-theme use across the policies. Reference to the health benefits of PA was most common (in over 80% of the policies), followed by social and community in approximately half of the policies, and the environment and wellbeing benefits both in around a third of the policies. Finally, reference to the educational benefits of PA was least common, identified in less than 20% of the policies.

**Table 1:** Social value domain and sub-theme presence in policy subsample.

Social value domain		Number of policies (out of 45)
Health		37 (82.2%)
Subtheme	Physical health	19
	Mental health	8
	Healthy urban planning	8
	Secondary economic	3
	Healthy ageing	3
Social & community		20 (44.4%)
Subtheme	Social ties	12
	Personal development	9
	Crime	7
	Active citizenship	3
	Cultural significance	3
Environment		15 (33.3%)
Subtheme	Active travel	10
	Eco-conscious attitudes	3
Wellbeing		15 (33.3%)
Education		8 (17.8%)

The main domain frequency should not necessarily equal the total of sub-theme frequencies. This is because main domain frequency includes policies which made general statements on the domain, which were not included in a sub-theme. The same policy may also reference multiple sub-themes, but is only counted once in the main domain figure.

Notably, holistic social value framing was lacking with only 2 out of the 45 policy documents (4.44%) recognising all five social value domains when discussing PA (see Supplementary Table S2). These were a Finnish environmental policy (Policy_E1) and a Swedish transport policy (Policy_T4). In other documents, certain sections reflected well-rounded social value framing of PA such as the Portuguese School Sports Programme 2017–2021, presenting PA “as a means of character building, health protection, environmental protection, social cohesion and inclusion” (Policy_E&Y1:11). Despite this, 60% of the policies referenced only two or fewer domains, with 10 documents referencing one or none.

Policies from the health and urban planning sectors in particular demonstrated the lowest holistic social value framing of PA. For example, four out of the seven documents from the health sector referenced only the health benefits of PA. By contrast, policies from the environmental sector contained the most comprehensive social value framing of PA, with half mentioning three or more domains. When examined by sector group, the health domain was most widely referenced across all sectors. The social and community domain was the next most recognised by the health, sport, education and youth and other sectors. By contrast, this was the environment domain for the environment, transport and urban planning sectors.

Finally, whilst the social value of PA was recognised, its economic quantification was only present in two policies; Policy_E1 from Finland (a financial proxy estimation of the social value gained from outdoor recreation) and Policy_T5 from Austria (SROI estimation related to active travel).

### Results by social value domain

#### Health

“Preserving and protecting health is a social responsibility, and one of the prerequisites for this is to increase physical activity” (Policy_H3:38)

Policies referencing the health domain discussed the importance of PA for fostering healthy lifestyles, with five sub-themes identified: physical health, mental health, healthy urban planning, secondary economic benefits, and healthy ageing. Reference to the physical health benefits of PA was most common, followed by reference to mental health and healthy urban planning (e.g., how active travel enables urban design with mixed land use layouts which minimise population exposure to pollutants, stress and disturbed sleep from traffic noise and facilitate engagement in active lifestyles). The fourth most widely acknowledged sub-theme in the health domain was the secondary economic benefits of PA, with the resultant boosted health status of individuals reducing health expenditure, absenteeism, and increasing workforce productivity. Finally, healthy ageing was the least present sub-theme, with only three policies (from Bulgaria, Hungary and Greece) recognising PA as an effective “long-term non-pharmaceutical” intervention to postpone and prevent age-related illness and increase the functional capacity of older populations for self-care (Policy_E4:147).

#### Social and community

“A strong sports movement means a stronger society” (Policy_S2:2)

Five sub-themes were identified for the social and community domain: social ties, personal development, crime, active citizenship, and cultural significance. Reference to social ties was most prevalent, with PA seen as supporting social cohesion and a sense of belonging. It was particularly recognised as helping marginalised individuals “overcome their social isolation” and as useful for student relations in school settings (Policy_S7:1). This was followed by personal development, relating to the promotion of skills such as creativity, teamwork, leadership, and enabling more holistic development beyond the school learning setting. The third most common sub-theme was crime, with PA recognised as helping reduce delinquency and criminal behaviours. It was seen as particularly useful for at-risk youths, providing a constructive sense of community and “directing them towards appropriate forms of engaging their free time” (Policy_S7:1). Some policies also referenced how pedestrianised areas and mixed-use neighbourhoods for active travel can increase a sense of security for users and decrease criminal activities. Finally, the sub-themes of active citizenship (e.g., sport volunteering and PA participation creating proactive citizenship behaviours) and the cultural significance of PA were the least present. Regarding culture, policies highlighted the ability of sport events to “connect and inspire people” as well as active tourism strengthening national identity (Policy_S3:2).

#### Environment

“Active mobility is the most energy-efficient, climate-friendly, resource-saving, healthy and safe way to get around, making it the most sustainable form of mobility there is” (Policy_T5:30)

Two clear sub-themes emerged for the environment domain. First, policies most commonly highlighted how active travel is important for “both people's health and the climate” (Policy_T5:55). Active travel, which includes walking and cycling, was framed as supporting sustainable lifestyle behaviours which decrease emissions, air and noise pollution, and support more space efficient transport infrastructures. Many policies also referenced the important contribution of PA to the Sustainable Development Goals (SDG), including SDG11 relating to cities, whereby active travel can support more sustainable urban infrastructure and neighbourhood design. Second, policies discussed how PA can foster eco-conscious attitudes. Three policies referenced how engagement can create a more environmentally aware society through stronger relationships with nature and awareness of climate issues. Sport events were also represented as opportunities to raise awareness of ecological issues and promote sustainable lifestyles. For example, Policy_E3 focused on the upcoming Paris 2024 Olympics as an opportunity to promote green energy use and environmentally conscious behaviours. It also proposed that high level sports players should use their visibility to promote “energy saving and eco-responsible behaviour” (Policy_E3:29).

#### Wellbeing

“we feel better and are happier with movement” (Policy_T4:150)

No clear sub-themes were distinguished in the wellbeing domain due to the variety of narratives identified. These included PA boosting quality of life, providing a sense of achievement, and being a source of fun and happiness. For example, one policy stated how PA “provides energy, fun, inspiration and meaning” to people's lives (Policy_S3:12).

#### Education

“physical activity stimulates the formation of new brain cells and therefore has a positive effect on learning ability” (Policy_T4:150)

Being the least prevalent domain, only eight papers referenced the educational benefits of PA. This was generally about how PA improves the quality of education, student attainment, concentration, and classroom behaviour to positively enhance learning ability. Furthermore, PA was recognised as important for improving peer relations to create a more positive learning environment and “engage young people who might be at risk of early school-leaving” (Policy_E&Y3:57).

## Discussion

This study aimed to assess the current use of social value framing of PA in policy. To the best of the researchers' knowledge, this is the first study of its kind, contributing to the knowledge gap of PA issue framing in public health. All five domains of social value were identified in policy documents, with variability in their usage. The health, and social and community benefits of PA were most widely acknowledged. Furthermore, results revealed variation in social value domain use between sectors. The health and social and community domains were more common among policies from the health and education and youth sectors, whilst the environment domain was more commonly referenced among the environment and urban planning sectors. While prioritising the domain native to the policy sector is both important and most natural, tying in more multifaceted social value framing with acknowledgement of wider co-benefits is of high utility for generating multisectoral action. The WHO publication on health for all policies (29) recognises cooperation between non-traditional or unexpected actors is important. To overcome the drivers of physical inactivity, increase motivation and construct a conducive social and physical environment for performing PA, it is essential to secure the engagement of multiple sectors (43). A lack of framing of PA in relation to wider social benefits, particularly those not usually recognised by a sector, may be limiting issue salience and current motivation for multisectoral action. Importantly, only few documents contained holistic social value framing referencing multiple domains.

The Covid-19 pandemic demonstrated the mutual importance of health and other sectors and drew attention to the utility of multisectoral collaboration (29). Moreover, the 2030 SDG Agenda focuses on long term collaboration, with PA related to many of its goals including SDG3 Health, SDG4 Education, SDG8 Economic Growth, SDG11 Sustainable Cities and SDG13 Climate Action (44). As such, the social value domains of health, social and community, and environment are likely to become increasingly impactful in the current political context [e.g., (39)]. The use of these three social value domains therefore holds potential for strategic framing of PA to boost action. The wellbeing domain could also be of interest given the growing prominence of the EU's Economy of Wellbeing Agenda and recognition of the interlinkages between health improvement, wellbeing and economic productivity (45). Moreover, this domain recognises the importance of individual factors, such as self-efficacy and motivation (10–12), for driving PA engagement, in interaction with the socio-environmental context. However, the wellbeing domain was the second least present domain in policy documents. This therefore acts as an area for future monitoring and research.

Heterogeneity was not only found in the frequency of appearance of the main social value domains but also in the specific sub-themes. Physical health was the most prevalent when discussing the health benefits of PA. It is relevant to highlight that, although mental health was the second most recognised sub-theme, its use lagged behind reference to physical health (inclusion in 8 vs. 19 policies respectively). Although research already indicates the mental health benefits of PA (46), it is important to increase scientific knowledge about this link. Within the social and community domain, there was also variability in reference to different sub-themes, with the most prevalent being social ties and the least being active citizenship and cultural significance. This may be due to the strong evidence base that participation in PA increases social capital (32). Finally, active travel was more commonly referenced when discussing the environmental benefits of PA, compared to the development of eco-conscious attitudes. Future studies could develop this line of research to understand how participation in PA can help promote such attitudes in the population. This is of additional significance given that recent research has revealed expert consensus on the need to further investigate the impact of PA and sport on the environment (47).

Additionally, SROI evidence may be a helpful tool for PA promotion given that it provides quantification of the social benefits of PA through a monetised figure. This is an important form of evidence for capturing policymaker attention (36). However, the use of SROI evidence was rare in this policy subsample. It is important to highlight existing concerns in literature regarding the methodological rigour of the SROI model due to the difficulty of quantifying intangible benefits (33). This challenge is evidenced by the wide variety of methods currently used to calculate the SROI ratio (34). The development of a robust, transparent SROI model applied to PA may therefore be useful to enhance the accuracy and credibility of such calculations and provide a common method to be used by different actors.

Sensitivity analysis identified that 10 EU countries (Belgium, Cyprus, Czechia, Italy, Latvia, Luxembourg, Malta, Romania, Slovakia, and Slovenia) were not represented in the final sample of documents. A reason for this could be that at least three policies were identified for each of the countries included in the subsample, with an average of 6.47. By contrast, only 1–5 policies were identified for the 10 non-included countries from the sensitivity analysis, with an average of 3. Moreover, the most recent policy per non-included country ranged from 2014 to 2020, in contrast to 2021–2022 for included countries. These findings might reflect variation in EU member state political focus on PA.

### Actionable recommendations

Based on the results from this research, policymakers are encouraged to engage multisectoral stakeholders in PA promotion. Social value framing could be a useful tool to support this, helping to evidence both the benefits of PA gained by a sector itself, alongside the co-benefits that it can bring across multiple sectors. As indicated by Kingdon's (23) multiple streams framework, issue framing is an important lever for achieving policy action. Social value framing of PA is therefore an area for policy improvement in order to highlight the wide co-benefits of PA, increasing issue salience and helping to generate multisectoral PA promotion. In the current political context, the health, environment and social and community social value domains are likely to be most important to prioritise in narratives around PA. To accompany this, efforts should be made to develop a unified, robust methodology for generating monetised SROI evidence to boost its credibility and help secure wider stakeholder engagement.

### Limitations

It is important to recognise several limitations of this study. Firstly, the policy search only involved WHO 2021 Physical Activity Factsheets and the EACEA National Policies Platform. Further publications may therefore exist which were not listed in these resources. National Ministry of Health websites were intended to be used as an additional search platform, but the difficulty of locating policies which referenced PA within such websites made this unfeasible given time and resource constraints. Secondly, a proportion of listed policies on these platforms could not be located as full-text documents. Thirdly, whilst DeepL is an advanced translation tool, the translation of policies may not be completely accurate. However, careful consideration was taken over selecting the most accurate tool currently available based on functionality, reviews, and comparative translation of extracts and this tool allowed the inclusion of policies not originally published in English. Fourthly, the involvement of a second reviewer to enable crosschecking of iterative coding would have been preferable and only a subsample of identified policies was analysed. However, saturation and sensitivity assessments were conducted to evaluate the comprehensiveness of the results.

## Conclusion

Given prevailing physical inactivity among the European population, it is important for meaningful policy change to occur. A shifted approach based on multisectoral action is central for increasing PA engagement and targeting the structural drivers of inactivity. Social value framing of PA could be of significant utility for this purpose, particularly for engaging a wider range of stakeholders in PA promotion. This study identified that social value framing of PA is used to a certain extent in existing EU member state policies. Variation was found in the comprehensiveness of social value framing of PA, with recognition of the health benefits of PA most widely established. Variation was also identified in holistic social value framing between sectors, with policies from the environmental sector recognising the widest range of co-benefits of PA whereas documents from the health sector demonstrated the most limited social value framing. The use of more holistic social value framing in policies which reference PA, recognising the benefits that extend beyond those accrued by a sector itself, could help to secure wider multisectoral stakeholder interest and therefore help to increase engagement in PA. Finally, the methodology used in this study could be applied in countries from other continents to evaluate the presence and utility of the social value framework in different locations and help promote PA globally.
