# Supplementary material for: Social value framing of physical activity in European Member State policies: a content analysis
Source: Front Sports Act Living. 2024 Jun 5;6:1415007. doi: 10.3389/fspor.2024.1415007 (PMC11188325; doi:10.3389/fspor.2024.1415007)
Supplement: Supplementary file 1 [file Table1.docx]

# Supplementary materials

Table S1. Final policy content analysis codebook.

| Main code | Subcodes | | | Description |
| --- | --- | --- | --- | --- |
| **HEALTH**  **(1)** | General   (1.1) | | | Reference to general improvement of health through PA, which does not fit into a sub-code |
|  | Healthy ageing   (1.2) | | | Reference to PA improving the health of older populations (e.g. – in those over 65yrs, reference to age-related conditions) |
|  | Mental health   (1.3) | | | Reference to PA improving mental health (e.g – anxiety, depression). Include reference to stress. |
|  | Physical health  (1.4) | | | Reference to PA improving physical health (e.g – NCDs, musculoskeletal injury risk, fitness) |
|  | Healthy urban planning   (1.5) | | | Reference to PA facilitating healthy urban planning practices (e.g – active transport, spatial planning, mixed use neighbourhoods) |
|  | Secondary economic  (1.6) | | | Reference to the improved health gained through PA engagement providing subsequent economic benefits (e.g – increased productivity, healthcare savings) |
| **SOCIAL & COMMUNITY**  **(2)** | General  (2.1) | | | Reference to general benefit of PA to communities and social connection, which doesn’t fit into a sub-code |
|  | Active citizenship   (2.2) | | | Reference to PA as fostering active citizenship behaviours (e.g – engaging with the community you live in) |
|  | Crime   (2.3) | | | Reference to PA reducing criminal, anti-social and violent behaviour |
|  | Cultural significance   (2.4) | | | Reference to the cultural importance of PA, both when participating and observing (e.g – for national identity) |
|  | Personal development   (2.5) | | | Reference to PA benefitting development, which doesn’t fit into a sub-sub-code |
|  |  | Child right  (2.5_1) | | Reference to PA as a right for all children |
|  |  | Emotional development  (2.5_2) | | Reference to the importance of PA for the development of social skills (e.g – self-control, teamwork, leadership) |
|  |  | Motor development  (2.5_3) | | Reference to the importance of PA for the development of physical skills (e.g – motor skill, balance, coordination). NB: This does not include physical development in terms of body growth, as this should go in the physical health code |
|  | Social ties  (2.6) | | | Reference to PA increasing social ties and community cohesion |
|  |  | Counters exclusion  (2.6.1) | | Specific reference to PA as counteracting social exclusion (e.g – inclusivity, reduced isolation) |
|  |  |  | Marginalised populations  (2.6.1_1) | Reference to social inclusion of specific marginalised populations through PA |
| **WELLBEING**  **(3)** | n/a | | | Reference to PA increasing happiness/quality of life/wellbeing of individuals |
| **EDUCATION**  **(4)** | n/a | | | Reference to PA improving educational quality and attainment |
| **ENVIRONMENT (5)** | General  (5.1) | | | Reference to the general benefit of PA to the environment, not fitting into a sub-code |
|  | Active travel  (5.2) | | | Reference to the environmental benefits of active travel (e.g – emission reduction) |
|  |  | Space efficient  (5.2.1) | | Reference to active transport as space efficient compared to roads and infrastructure required for motorised forms, hence benefits urban design |
|  | Eco-conscious attitudes  (5.3) | | | Reference to PA increasing awareness of environmental issues and fostering eco-conscious attitudes |
|  |  | | |  |
| Additional information  (6) | Other  (6.1) | | | Code to gather sections of text which do not relate to the specific social value domains, but may still be of interest |
|  | Future directions  (6.2) | | | References to future directions for action to increase PA (incl. impact of COVID and opportunities it has now created) |
|  | General statements  (6.3) | | | General statements on the ‘benefit of PA’, which do not fit into a main code |
|  | Multisectoral  (6.4) | | | Reference to a need for multisectoral action for health improvement in general |
|  |  | PA related  (6.4.1) | | Reference to need for multisectoral action to increased engagement in PA specifically |

**Table S2. Included policies**

Please note, no shading indicates the document was included in the original subsample. Documents in light grey were included through saturation assessment.

| Domain | ID | Country | Publication date | Title | Source |
| --- | --- | --- | --- | --- | --- |
| Health | Policy_H1 | Portugal | 2021 | National Programme for Physical Activity Promotion  [PROGRAMA NACIONAL PARA A PROMOÇÃO DA ATIVIDADE FÍSICA] | EACEA |
|  | Policy_H2 | Bulgaria | 2021 | National Health Strategy 2021-2030  [НАЦИОНАЛНА ЗДРАВНА СТРАТЕГИЯ  2021 - 2030] | WHO |
|  | Policy_H3 | Hungary | 2021 | Healthy Hungary 2021-2027: Health Sector Strategy  [EGÉSZSÉGES MAGYARORSZÁG 2021−2027: EGÉSZSÉGÜGYI ÁGAZATI STRATÉGIA] | EACEA |
|  | Policy_H4 | Ireland | 2021 | Healthy Ireland: Strategic Action Plan 2021-2025 | WHO |
|  | Policy_H5 | Poland | 2021 | Regulation on the National Health Programme 2021-2025  [PW SPRAWIE NARODOWEGO PROGRAMU ZDROWIA NA LATA 2021–2025] | WHO |
|  | Policy_H6 | Finland | 2021 | Promoting wellbeing, health and security 2030 Government Resolution   [HYVINVOINNIN, TERVEYDEN JA TURVALLISUUDEN EDISTÄMINEN 2030] | WHO |
|  | Policy_H7 | Croatia | 2020 | Action Plan for the Prevention and Control of Chronic Non-communicable Diseases 2020-2026   [AKCIJSKI PLAN ZA PREVENCIJU I NADZOR NAD KRONIČNIM NEZARAZNIM BOLESTIMA 2020. - 2026.] | WHO |
|  |  |  |  |  |  |
| Sport | Policy_S1 | Spain | 2022 | Basic sport support plan 2030   [ADB2030: APOYO AL DEPORTE BASE] | EACEA |
|  | Policy_S2 | Sweden | 2022 | Strategic plan for sport 2022-2025  [STRATEGISK PLAN FÖR IDROTTSRÖRELSEN] | WHO |
|  | Policy_S3 | Netherlands | 2022 | National sports agreement  [NATIONAAL SPORTAKKOORD] | WHO |
|  | Policy_S4 | Bulgaria | 2021 | National strategy for the development of physical education and sport in the Republic of Bulgaria 2012-2022  [НАЦИОНАЛНА ПРОГРАМА ЗА РАЗВИТИЕ НА ФИЗИЧЕСКАТА АКТИВНОСТ, ФИЗИЧЕСКОТО ВЪЗПИТАНИЕ, СПОРТА И СПОРТНО-ТУРИСТИЧЕСКАТА ДЕЙНОСТ 2021 – 2022 г.] | WHO |
|  | Policy_S5 | Estonia | 2021 | Estonian Sport Policy Until 2030   [EESTI SPORDIPOLIITIKA PÕHIALUSTE AASTANI 2030] | WHO |
|  | Policy_S6 | Bulgaria | 2020 | Programme for the development of sport for people with disabilities for 2020  [ПРОГРАМА ЗА РАЗВИТИЕ НА СПОРТА ЗА ХОРА С УВРЕЖДАНИЯ за 2020 г] | EACEA |
|  | Policy_S7 | Bulgaria | 2020 | Sport for children at risk for 2020  [ПРОГРАМА „СПОРТ ЗА ДЕЦА В РИСК“ за 2020 г] | EACEA |
|  |  |  |  |  |  |
| Environment | Policy_E1 | Finland | 2022 | National Strategy for Nature Recreation 2030   [KANSALLINEN LUONNON VIRKISTYSKÄYTÖN STRATEGIA 2030] | WHO |
|  | Policy_E2 | Hungary | 2022 | National Active Tourism Strategy 2030  [NEMZETI AKTÍV TURISZTIKAI STRATÉGIA] | WHO |
|  | Policy_E3 | France | 2022 | Plan for Energy Sobriety in Sport  [PLAN DE SOBRIÉTÉ ÉNERGÉTIQUE DU SPORT] | EACEA |
|  | Policy_E4 | Greece | 2021 | National plan for accessibility, with a focus on climate change   [ΕΘΝΙΚΟ ΣΧΕΔΙΟ ΓΙΑ ΤΗΝ ΠΡΟΣΒΑΣΙΜΟΤΗΤΑ ΜΕ ΕΜΦΑΣΗ ΣΤΗΝ ΚΛΙΜΑΤΙΚΗ ΑΛΛAΓΗ-ΚΛΙΜΑΤΙΚΗ ΚΡΙΣΗ] | WHO |
|  | Policy_E5 | France | 2021 | 4th national environmental health plan   [4e PLAN NATIONAL SANTÉ ENVIRONNEMENT] | WHO |
|  | Policy_E6 | Greece | 2019 | National Energy and Climate Plan | WHO |
|  | Policy_E7 | Finland | 2017 | Healthy Parks, Healthy People: Health and wellbeing 2025 programme | WHO |
|  | Policy_E8 | Sweden | 2005 | Update 2005: Progress towards Sweden’s environmental objectives in the county of Stockholm | WHO |
|  |  |  |  |  |  |
| Transport | Policy_T1 | Germany | 2022 | National Cycling Plan 3.0 | WHO |
|  | Policy_T2 | Sweden | 2022 | Transport Policy Goals: Summary Report | WHO |
|  | Policy_T3 | Sweden | 2022 | VGU Guides: Design of roads and streets   [VGU-GUIDEN VÄGARS OCH GATORS UTFORMNING] | WHO |
|  | Policy_T4 | Sweden | 2022 | Traffic for an attractive city   [TRAFIK FÖR EN ATTRAKTIV STAD UNDERLAG TILL HANDBOK] | WHO |
|  | Policy_T5 | Austria | 2021 | Austria's 2030 Mobility Master Plan | WHO |
|  | Policy_T6 | Lithuania | 2021 | Improving energy efficiency and renewable energy, centralised use of resources, financial measures to be implemented in the heating, cooling and transport sectors: Preliminary assessment  [ENERGIJOS VARTOJIMO EFEKTYVUMO  DIDINIMO IR ATSINAUJINANČIŲ ENERGIJOS  IŠTEKLIŲ NAUDOJIMO CENTRALIZUOTO ŠILUMOS IR VĖSUMOS TIEKIMO BEI  TRANSPORTO SEKTORIUOSE PLANUOJAMŲ  ĮGYVENDINTI FINANSINIŲ PRIEMONIŲ IŠANKSTINIS VERTINIMAS] | WHO |
|  | Policy_T7 | Denmark | 2020 | Government agreement on the green transition of road transport  [AFTALE OM GRØN OMSTILLING AF VEJTRANSPORTEN] | WHO |
|  |  |  |  |  |  |
| Education & youth | Policy_E&Y1 | Portugal | 2017 | School sports programme 2017-2021   [PROGRAMA DO DESPORTO ESCOLAR 2017 - 2021] | EACEA |
|  | Policy_E&Y2 | Hungary | 2016 | Changing the speed in higher education 2016-2020  [FOKOZATVÁLTÁS A FELSŐOKTATÁSBAN KÖZÉPTÁVÚ SZAKPOLITIKAI STRATÉGIA 2016-2020] | WHO |
|  | Policy_E&Y3 | Ireland | 2014 | Better outcomes brighter futures : The national policy framework for children & young people 2014-2020 | EACEA |
|  | Policy_E&Y4 | Poland | 2014 | Youth activity programme 2015-2016   [RZĄDOWY PROGRAM AKTYWNOŚCI SPOŁECZNEJ MŁODZIEŻY NA LATA 2015-2016] | EACEA |
|  | Policy_E&Y5 | Austria | 2021 | Celebration of 10 years of the Child and Youth Health Strategy  [FESTVERANSTALTUNG 10 JAHRE KINDER – UND JUGENDGESUNDHEITSSTRATEGIE] | EACEA |
|  | Policy_E&Y6 | Ireland | 2007 | Teenspace: National recreation policy for young people | EACEA |
|  |  |  |  |  |  |
| Urban planning | Policy_U1 | Sweden | nd | National guidelines for green structure planning   [GRÖN INFRASTRUKTUR I FYSISK PLANERING] | WHO |
|  | Policy_U2 | Sweden | 2018 | Strategy for living cities – a policy for sustainable urban development  [SKRIVELSENS HUVUDSAKLIGA INNEHÅLL ] | WHO |
|  | Policy_U3 | Lithuania | 2011 | Streets and Local Roads. General Requirements  [ĮSAKYMAS DĖL STATYBOS TECHNINIO REGLAMENTO STR 2.06.04:2014 „GATVĖS IR VIETINĖS REIKŠMĖS KELIAI. BENDRIEJI REIKALAVIMAI“ PATVIRTINIMO] | WHO |
|  | Policy_U4 | Sweden | 2010 | Planning and building act 2010 | WHO |
|  |  |  |  |  |  |
| Other | Policy_O1 | Croatia | 2020 | The national development strategy Croatia 2030 | WHO |
|  | Policy_O2 | Poland | 2020 | Strategy for the development of human capital 2030   [STRATEGIA  ROZWOJU  KAPITAŁU LUDZKIEGO  2030] | WHO |
|  | Policy_O3 | Poland | 2020 | Active forms of countering social exclusion – new dimensions 2020  [AKTYWNE FORMY PRZECIWDZIALANIA WYKLUCZENIU SPOLECZNEMU - NOWY WYMIAR 2020] | WHO |
|  | Policy_O4 | Denmark | 2019 | Overview of 'inclusion in local communities through physical education' project pool   [OVERSIGT OVER PROJEKTER I PULJEN ’INKLUSION I LOKALE FÆLLESSKABER GENNEM IDRÆT] | WHO |
|  | Policy_O5 | Sweden | 2018 | Sweden’s work on global health – implementing the 2030 Agenda | WHO |
|  | Policy_O6 | Portugal | 2017 | Intersectoral commission for the promotion of physical activity [Despacho n.º 3632/2017] | WHO |
